# Heart Rate and Risk of Cancer Death in Healthy Men

**DOI:** 10.1371/journal.pone.0021310

**Published:** 2011-08-03

**Authors:** Xavier Jouven, Sylvie Escolano, David Celermajer, Jean-Philippe Empana, Annie Bingham, Olivier Hermine, Michel Desnos, Marie-Cécile Perier, Eloi Marijon, Pierre Ducimetière

**Affiliations:** 1 INSERM PARCC University Paris Descartes UMR-S970, Paris, France; 2 AP-HP HEGP, Paris, France; 3 Sydney Medical School, University of Sydney, Sydney, Australia; 4 Department of Adult Hematology CNRS UMR 8147, Hopital Necker Enfants-Malades, University Paris Descartes, AP-HP, Paris, France; 5 AP-HP, HEGP, Department of Cardiology, University Paris V, Paris, France; 6 IFR 69, INSERM, Université Paris Sud-XI, Villejuif, France; University of Ottawa, Canada

## Abstract

**Background:**

Data from several previous studies examining heart-rate and cardiovascular risk have hinted at a possible relationship between heart-rate and non-cardiac mortality. We thus systematically examined the predictive value of heart-rate variables on the subsequent risk of death from cancer.

**Methods:**

In the Paris Prospective Study I, 6101 asymptomatic French working men aged 42 to 53 years, free of clinically detectable cardiovascular disease and cancer, underwent a standardized graded exercise test between 1967 and 1972. Resting heart-rate, heart-rate increase during exercise, and decrease during recovery were measured. Change in resting heart-rate over 5 years was also available in 5139 men. Mortality including 758 cancer deaths was assessed over the 25 years of follow-up.

**Findings:**

There were strong, graded and significant relationships between all heart-rate parameters and subsequent cancer deaths. After adjustment for age and tobacco consumption and, compared with the lowest quartile, those with the highest quartile for resting heart-rate had a relative risk of 2.4 for cancer deaths (95% confidence interval: 1.9–2.9, p<0.0001) This was similar after adjustment for traditional cardiovascular risk factors and was observed for the commonest malignancies (respiratory and gastrointestinal). Similarly, significant relationships with cancer death were observed between poor heart rate increase during exercise, poor decrease during recovery and greater heart-rate increase over time (p<0.0001 for all).

**Interpretation:**

Resting and exercise heart rate had consistent, graded and highly significant associations with subsequent cancer mortality in men.

## Introduction

Heart-rate plays a central role in the cardiovascular system and depends on the balance between vagal and adrenergic tone. Higher levels of resting heart-rate have been associated with total and especially cardiovascular mortality in numerous studies [1]–[22]. The pattern of heart-rate increase during exercise and decrease during recovery has also been associated with all-cause mortality by several authors, [6]–[8], [11], [16] with cardiovascular mortality [11], [15], [16] and sudden cardiac death[11] by others. Some studies involved cardiovascular patients usually referred for an exercise test, [6]–[8], [15], [16] whereas others involved the general population [11].

Interestingly, several of these studies have shown little if any difference between the relative risk for cardiovascular death compared with total mortality, associated with higher resting heart rate [23]. For example, an association between resting heart-rate and non cardiovascular mortality (relative risk = 1.16, confidence interval = 1.13–1.19) was noted in 379,843 middle-aged men and women from Norway, similar to the 1.10 (CI:1.05–1.15) for cardiovascular and 1.14 (CI:1.11–1.17) for total death, but as stated by the authors: “since it was not the focus of the study, this finding was not pursued” [22]. Such data suggest the possibility that higher resting heart rate may be equally associated with non cardiovascular death, the majority of which would be expected to be due to cancer in developed countries. Few data are available however to examine the relationships between heart-rate variables and cancer mortality. Although an association between a high level of heart-rate and cancer mortality was hypothesized 30 years ago in two of three epidemiologic studies from Chicago[24], this possible association has not been comprehensively examined.

The purpose of the present work was thus to examine specifically and prospectively the potential associations between different heart-rate parameters and various causes of cancer mortality, in a well characterized general population. The Paris Prospective Study I gave us a unique opportunity to address these issues as not only resting heart-rate but also the heart-rate pattern during exercise, recovery and the heart-rate change over 5 years were measured in the same study, and different causes of mortality were followed up over more than 25 years.

## Methods

Details of the Paris Prospective Study 1 concerning the recruitment, design, and procedures have been described previously [11], [25]. Briefly, a consecutive examination of 7746 native Frenchmen employed by the Paris Civil Service, aged 42 to 53 years, was carried out from 1967 to 1972. That sample consisted of 93.4 percent of the total number of employees in early 1967 who were born between 1917–1928. Subjects had electrocardiograms and physical examinations conducted by a physician, provided blood samples for laboratory tests, and answered questionnaires administered by trained interviewers. Resting heart-rate was clinically determined by measurement of the radial pulse during a one-minute recording, after five-minutes rest in the supine position. Diabetic status was defined as past or present reported diabetes, whether treated or not. Tobacco consumption was assessed by questionnaires and reported as the average use in grams per day over the 5 years preceding the study. Current physical activity applied to subjects who performed more than one hour of regular activity per week.

### Exercise test protocol

The standardized protocol of the bicycle exercise test performed has been described previously [11]. It consisted of 3 successive work loads: 2 min at 82 W, 6 min at 164 W and the last 2 min at 191 W for a maximal 10-min test duration. There was no cool-down period. Cardiac rhythm was continuously monitored, and a bipolar lead (V5 and V5R) was recorded at rest and for 30 sec every 2 min during exercise, at maximal effort and every 1 min during the 10-min recovery time, or whenever the monitoring physician observed an arrhythmia. Heart-rate was measured at rest, before exercise, every two minutes during exercise, at peak exercise and every minute during recovery. Testing was terminated because of fatigue, dyspnea, leg discomfort, any chest pain, systolic blood pressure>250 mm Hg, heart-rate ≥ 180 beats/min, ventricular tachycardia or ischemic electrocardiographic changes. Exercise testing was carried out in 6565 men, with complete data available for 6456.

### Heart-rate measurements

In addition to resting heart-rate, three other heart-rate parameters were defined as being of potential significance. Firstly, the heart-rate increase during exercise was the difference between the maximum heart-rate achieved at peak exercise and resting heart-rate [11]. Secondly, the heart-rate during recovery was the difference between maximum heart-rate achieved at maximum exercise and heart-rate recorded one minute later [11]. Thirdly, heart-rate change after 5 years was the difference between resting heart-rate recorded at baseline and 5 years later, available in the 5139 men who completed the 5 year follow-up examination [12].

### Follow-up

Until retirement (official age 65 years), the administrative department in charge of the study population provided a list of deceased subjects annually. All available data relevant to the causes of death were collected from specific inquiries, i.e. medical records from hospital departments or general practitioners indicated by the relatives of the deceased. The data were then reviewed by an independent medical committee. After retirement (usually after age 65), causes of death were obtained from death certificates. The eighth and ninth revisions of the International Classification of Diseases [26] were used for coding. The end of the follow-up period was January 1, 1994. Vital status could not be determined for 355 subjects (4.6 percent). Their characteristics at baseline and during exercise were not significantly different from the remaining 6101 men studied.

### Statistical methods

All statistical analyses were performed using SAS 9.1.3 (Cary, Nc). Hazards ratios (HRs) of each heart rate parameter for each study outcome were estimated in separate Cox proportional hazard models. Multivariate-adjusted HRs on known cancer risk factors (age and tobacco consumption) were quantified. For the purpose of comparison, mortality risks were assessed in a second model for traditional cardiovascular risk factors (age, body mass index, systolic blood pressure, diabetes mellitus, tobacco consumption, current physical activity and blood level of cholesterol). In all analyses, each heart-rate parameter was divided in quartiles and the first quartile (the lowest value) was taken as the reference category.

The results of the survival analyses were also represented graphically using Cox multivariate survival model of proportional hazard assumption. In each graph, we plotted the predicted survival curves for each of the quartiles of heart-rate.

When the evolution of heart-rate after 5 years was entered into the model, the statistical analysis was restricted to the 5,139 subjects for whom that variable was available. Additional sensitivity analyses were carried out (i) excluding causes of death assessed from death certificates only, (ii) after exclusion of the 48 cancer deaths which occurred in the first 5 years after baseline study, to exclude the possibility that resting or exercise related heart-rate might have been related to undiagnosed malignancy at the time of baseline visit, and (iii) after splitting smoking-related and other cancers.

## Results

Among the 6101 men, and after 25 years of follow-up representing over 150 000 person years of observation, 1635 deaths were recorded, with 435 cardiovascular deaths and 1200 non cardiovascular deaths. These included 771 deaths due to cancer, with 241 deaths of pulmonary origin, (including 178 of trachea, bronchus and lung and 43 of larynx) 210 of gastrointestinal origin (including 67 of colon and rectum, 42 of oesophagus, 27 of stomach, 32 of liver, and 32 of pancreas), 89 of genitourinary origin (including 44 of prostate and 25 of bladder) and 231 miscellaneous (including 108 of unspecified sites, 53 of lymphatic and hematopoietic tissue, and 9 of unspecified nature). The baseline characteristics of subjects by mortality causes are shown in table 1. Those who died from cancer had the highest level of tobacco consumption whereas those who died from cardiovascular causes had the highest body mass index, systolic blood pressure, cholesterol level and the highest proportion of diabetes mellitus.

**Table 1:** Baseline characteristics of the 6101 men according to causes of mortality.

	Mortality								
	All-cause mortality	Cardio vascular mortality	Non cardiovascular mortality	Cancer mortality					Alive
				all	respiratory	digestive	genito-urinary	other or unknown	
	(n = 1635)	(n = 435)	(n = 1200)	(n = 771)	(n = 241)	(n = 210)	(n = 89)	(n = 231)	(n = 4466)
Age (y)	48.0 (1.8)	48.0 (1.8)	48.0 (1.8)	48.0 (1.8)	47.9 (1.9)	48.1 (1.8)	48.3 (1.7)	47.8 (1.7)	47.5 (2.0)
Body-mass index (kg/m^2^)	25.7 (3.5)	26.1 (3.3)	25.5 (3.7)	25.4 (3.6)	24.7 (3.4)	26.1 (3.7)	26.0 (3.4)	25.3 (3.6)	25.8 (3.0)
Systolic blood pressure (mmHg)	14.9 (2.1)	15.0 (2.1)	14.8 (2.1)	14.7 (2.0)	14.6 (2.1)	14.9 (2.0)	14.7 (2.0)	14.8 (2.1)	14.1 (2.0)
Total cholesterol (mg/dl)	223 (45)	235 (47)	219 (44)	218 (42)	217 (42)	221 (44)	219 (40)	217 (41)	222 (41)
Tobacco consumption (g/day)	15.2 (10.7)	14.1 (10.1)	15.6 (10.8)	16.8 (10.6)	20.6 (9.5)	14.5 (11.2)	13.4 (9.1)	16.4 (10.8)	10.5 (10.2)
Leukocytes (×10^9^ units/l)	6.8 (2.0)	6.8 (1.9)	6.7 (2.1)	6.9 (2.1)	7.2 (2.0)	6.6 (1.9)	6.4 (1.8)	7.0 (2.4)	6.3 (1.8)
Hemoglobin (g/l)	15.1 (1.0)	15.1 (1.0)	15.1 (1.0)	15.1 (1.0)	15.1 (1.1)	15.1 (1.0)	15.2 (0.9)	15.1 (1.0)	15.1 (0.9)
Current physical activity	10.7%	13.4%	9.7%	10.3%	10.1%	9.2%	10.5%	11.2%	16.5%
Diabetes mellitus	1.5%	1.6%	1.5%	1.3%	0.8%	1.9%	0%	1.8%	1.0%

Continuous data are given as mean (standard deviation)

Tobacco consumption is the average consumption (grams per day) in the five years preceding the study.

Physical activity applies to subjects who performed more than one hour of regular activity per week.

The Paris Prospective Study I.

Subjects were divided into quartiles according to the level of resting heart-rate (interquartile values in beats per min: 60, 67 and 73); of heart-rate increase during exercise (interquartile values in beats per min: 85, 99 and 109); of heart rate decrease after one minute recovery (interquartile values in beats per min: 25, 32 and 39); and of heart-rate change after 5 years (interquartile values in beats per min: −8, −1, and +5).

The age and tobacco-adjusted hazard ratios of mortality for the highest quartiles of each heart-rate parameter are reported in table 2. As expected, the heart-rate pattern at rest, during exercise and recovery had a graded and significant association with total and cardiovascular mortality.

**Table 2:** Age and tobacco adjusted relative risks of death according to quartiles of heart-rate parameters.

		Mortality Relative Risk (95% CI)					
	quartile	All-cause mortality (n = 1635)	Cardio vascular mortality (n = 435)	Non cardio vascular mortality (n = 1200)	All-cause cancer mortality (n = 771)	Respiratory cancer mortality (n = 241)	Digestive cancer mortality (n = 210)
Resting HR	2^nd^	1.3 [1.2;1.6]	1.4 [1.0;1.8]	1.3 [1.1;1.6]	1.6 [1.2;2.0]	1.8 [1.2;2.7]	1.6 [1.0;2.5]
	3^ rd^	1.5 [1.3;1.8] ***	1.6 [1.2;2.1] ***	1.5 [1.3;1.8] ***	1.6 [1.3;2.0] ***	1.5 [1.0;2.3] ***	1.6 [1.1;2.5] ***
	4^ th^	2.1 [1.8;2.4]	2.0 [1.5;2.7]	2.1 [1.8;2.5]	2.4 [1.9;2.9]	3.0 [2.1;4.5]	2.3 [1.5;3.3]
ΔHRexercise	2^nd^	1.3 [1.1;1.5]	1.8 [1.3;2.3]	1.1 [1.0;1.4]	1.1 [0.9;1.4]	0.9 [0.6;1.4]	1.1 [0.7;1.6]
	3^ rd^	1.6 [1.4;1.8] ***	2.1 [1.6;2.8] ***	1.4 [1.2;1.7] ***	1.4 [1.1;1.7] ***	1.5 [1.0;2.2] ***	1.1 [0.8;1.7]
	4^ th^	1.7 [1.5;2.0]	2.3 [1.8;3.0]	1.6 [1.4;1.9]	1.6 [1.3;1.9]	2.0 [1.4;2.9]	1.4 [0.9;2.0]
ΔHRrecovery	2^nd^	1.1 [1.0;1.3]	1.0 [0.7;1.3]	1.2 [1.0;1.4]	1.1 [0.9;1.4]	1.3 [0.8;1.9]	1.2 [0.8;1.8]
	3^ rd^	1.0 [0.9;1.2] ***	0.9 [0.7;1.2]	1.1 [0.9;1.3] ***	1.0 [0.8;1.3] ***	1.1 [0.7;1.6] **	1.0 [0.7;1.6]
	4^ th^	1.4 [1.3;1.6]	1.3 [1.0;1.6]	1.5 [1.3;1.8]	1.5 [1.2;1.8]	1.8 [1.3;2.5]	1.4 [1.0;2.0]
ΔHR5year §	2^nd^	1.1 [0.9;1.4]	0.8 [0.5;1.2]	1.2 [1.0;1.6]	1.4 [1.0;1.8]	1.5 [0.9;2.4]	1.3 [0.8;2.2]
	3^ rd^	1.2 [1.0;1.4] ***	1.1 [0.7;1.5]	1.2 [1.0;1.5] ***	1.1 [0.9;1.5] **	1.1 [0.7;1.9]	1.2 [0.7;2.0]
	4^ th^	1.7 [1.4;2.0]	1.2 [0.9;1.8]	1.9 [1.5;2.3]	1.6 [1.2;2.2]	1.7 [1.0;2.9]	1.4 [0.8;2.4]

ΔHRexercise: difference between heart-rate at peak exercise and resting heart-rate

ΔHRrecovery: difference between heart-rate at peak exercise and after 1 min recovery

ΔHR5year: difference between resting heart-rate recorded at year 5 and at baseline

§ The analysis is restricted to 5139 subjects, see methods

Relative risk that was associated with a heart-rate measurement is given for the second, third and fourth quartile, taken the first quartile as reference. Relative risks were estimated with the Cox proportional-hazard model. CI denotes confidence interval.

Symbols of p-values for trend test: ***: p<0.0001; **: p<0.01

The Paris Prospective Study I.

A graded and increased risk for noncardiovascular and cancer mortality was observed with (i) a higher resting heart-rate, (ii) a poorer heart-rate increase during exercise and (iii) a slower decrease of heart-rate during recovery, although the relationship did not always reach statistical significance for the last. For example, the relative risk of cancer death for subjects in the highest compared with the lowest quartile for resting heart rate was 2.4 (1.9–2.9, p<0.001). Higher heart-rate increase after 5 years was also predictive of subsequent cancer mortality. Analysis by cancer causes (gastrointestinal and respiratory) yielded similar trends as for death from overall cancer, although statistical significance was generally not reached.

**Figure 1:**
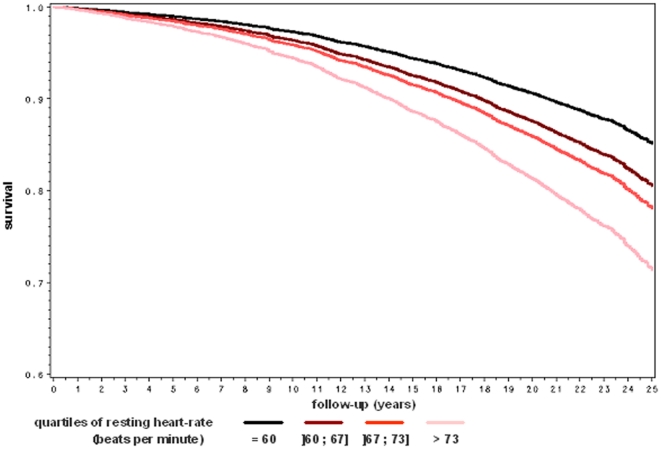
Multivariate adjusted overall survival rate by quartile of resting heart-rate. The Paris Prospective Study I.

**Figure 2:**
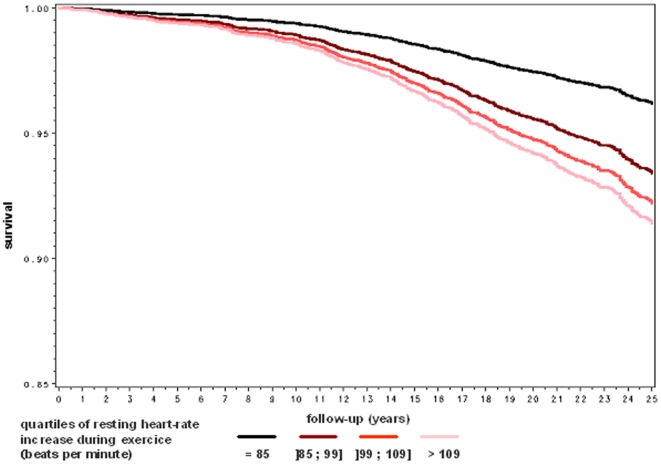
Multivariate adjusted cardiovascular survival rate by quartile of heart-rate increase during exercise. The Paris Prospective Study I.

**Figure 3:**
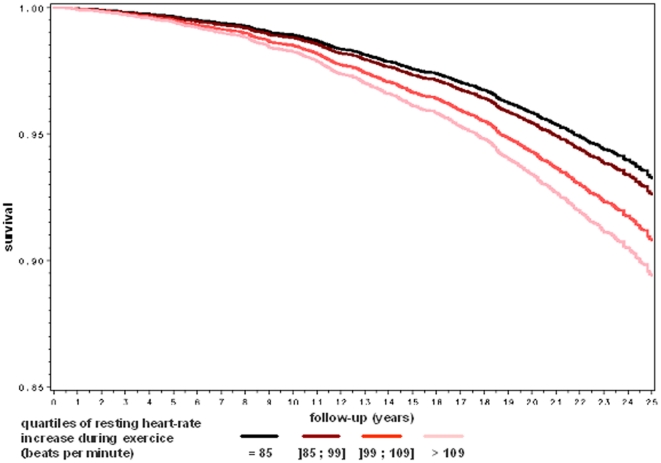
Multivariate adjusted cancer free survival rate by quartile of heart-rate increase during exercise. The Paris Prospective Study I.

A series of survival curves by quartile of heart rate parameters are plotted in figures 1, 2 and 3 after adjustment for age, body mass index, systolic, diabetes mellitus, tobacco consumption, current physical activity and blood level of total cholesterol. As shown in figure 1, the overall survival rate decreased significantly by quartile of resting heart-rate. As shown in figures 2 and 3, the pattern of the relationship between heart-rate increase during exercise and mortality was similar for cardiovascular and cancer mortality.

### Sensitivity analyses

When additional adjustments were made for baseline haemoglobin level or white blood cell count, the results were unchanged. Additional analyses were carried out excluding causes of death assessed from death certificates only and after exclusion of the 48 cancer deaths occurred in the first 5 years after baseline, and similar results were obtained. Finally, exclusion of the 271 subjects with an ischemic response to exercise or of the 117 who had an impaired chronotropic response (i.e. those who did not achieve 80 percent of the predicted maximal heart-rate defined as 220 beats per minute minus age), also gave similar results. Similar results were observed in both groups when cancer deaths were split into smoking-related (n = 306) and other cancers (n = 465).

## Discussion

In this prospective study of over 6000 asymptomatic middle-aged men selected from a general population and followed for mortality over 25 years, we found a continuous, graded and highly significant association between increased heart rate and increased risk of death from cancer. Our data confirmed the previously reported association between increased heart rate and cardiovascular deaths [1]–[22]. The major unexpected finding, however, was that the magnitude of the heart-rate related risks were similar between cardiovascular deaths and deaths from cancers. These significant associations remained important and comparable, after adjustment for traditional cardiovascular risk factors.

These findings appear robust as they persisted after exclusion of deaths that occurred during the initial 5 years of follow-up (possibly related to the existence of a disease already present at inclusion although not clinically detectable) or deaths after age 65 (when causes of deaths were obtained from death certificates rather than specific contemporaneous family and medical inquiries). Furthermore, these associations between cancer mortality and heart-rate appear credible and consistent, as they were observed for all four of the measured heart-rate parameters (resting, exercise, recovery and change over 5 years).

Exercise tests are mainly indicated in the cardiovascular field to detect subclinical ischemic heart disease and/or ventricular arrhythmias. The heart-rate pattern during exercise and recovery has been shown to be strongly predictive of cardiovascular and all-cause mortality. [6]–[8], [11], [15], [16]. By contrast, data concerning heart-rate and cancer mortality risk are much more sparse. An association between a high level of resting heart-rate and cancer mortality was first hypothesized in 1981, when Persky et al. published three observational studies together, concerning middle-aged men from Chicago [24]. In this paper, a significant association was noted in two but not in the third study, however in one of the “positive” studies, the association was no longer significant when deaths in the first two years of follow-up where excluded (suggesting the possibility of subclinical cancer already being present in some of the tachycardic subjects). Resting heart-rate was the only heart-rate parameter measured.

In a more extensive follow-up of these Chicago studies published in 1999 and involving men and women of different ages, Greenland et al. found a weak but significant association between heart-rate and subsequent cancer mortality in middle-aged men and women (relative risk 1.20, CI: 1.11 to 1.28 in men and 1.15, CI: 1.05 to 1.26 in women) but not in young or older age groups [27]. Similarly, Thomas et al found a graded relationship between heart-rate and cancer risk after 8 years follow-up in a large group of middle-aged men, [23] however in both of these studies only one heart-rate parameter (resting) was studied, on only one single occasion. [23], [27] More recently Kristal Boneh et al. reported a lack of association between resting heart-rate and cancer deaths, but young to middle-aged men were followed for only 8 years and there were only 45 cancer deaths recorded [21]. Thus previously published work has been somewhat inconsistent and inconclusive, regarding a possible relationship between heart-rate and cancer.

With the exceptions of tobacco exposure for lung cancer and familial history for colorectal malignancy, there have been few risk factors associated with cancer death which confer as substantial a relative risk, as did heart-rate in the present study [28]–[29].

The mechanisms that could explain the association between heart-rate and cancer mortality are unclear. Is heart rate a marker of risk for developing cancer, or a marker of risk of dying if one individual develops cancer?

One possibility is that heart-rate increase might be a marker of chronic stress and anxiety, which may in turn be related to an increased risk for cancer. Recent data suggest that the link between chronic stress and cancer might be related to genetic instability as a consequence of telomere length reduction and telomerase activity decrease [30].

Another possibility is that low-grade inflammation which is present in certain malignant conditions may play a role although it is not known to what extent such inflammation might be associated with heart-rate increase. C-reactive protein measurements were not available at baseline (1967–72) in the present study, however when additional adjustments were made for white blood cell count (reflecting to some extent the inflammatory process), our results were unchanged. Similarly, subclinical anaemia is an unlikely explanation, as adjusting for baseline haemoglobin did not alter the strengths of associations observed.

It is tempting to speculate a potential role for autonomic imbalance. Heart-rate is a general integrator of many physiologic systems such as cardiovascular, respiratory and neuropsychologic. An increase in heart-rate is generally interpreted as mediated by an imbalance between vagal and adrenergic tone [31], which may in turn be related to cancer risk. We could envisage the autonomic nervous system to be stimulated by the development of malignant cells in a given tissue. We could also envisage that subjects with pre-existing disturbance of their autonomic system may have a lower immunity defense system and an increased risk of dying if the individual develops cancer. Further works are clearly needed to address these important issues.

In any event, it seems likely that heart-rate is an epiphenomenon whereby an underlying factor separately causes heart-rate increase and cancer risk, although a direct link cannot be confidently excluded.

The present results emphasise the potential prognostic value of heart-rate and suggest a need to standardize its recording. Heart-rate measurement is inexpensive and non-invasive. If confirmation of these results is provided from other studies, routine heart-rate measurements at rest and on exercise may be worth considering in cancer risk prediction algorithms. Our results however, do not suggest a “threshold” level of risk above a certain specific heart-rate, rather a continuous and progressive relationship with cancer risk.

There are limitations of this study. Certain important cancer risk factors were not recorded, such as family history of colorectal cancer or exposure to environmental tobacco smoke in non-smokers. These were not clearly appreciated as risk factors at the time of the baseline heart-rate examinations (1967–72). These factors would seem unlikely, however, to have explained the significant heart-rate relationships observed. Furthermore, only men were studied and thus the results cannot be considered applicable to women. Self-report was relied upon for tobacco and alcohol consumption. Deaths over age 65 years were categorized by death certificates only, with causes of death not confirmed by specific additional enquiries. However, a systematic confounding of our results appears unlikely, especially as the associations were of similar magnitude when certificated deaths (over age 65 years) were excluded from the analyses.

As a long-term cohort study, there have been changes in exposure since baseline assessment that could be related to cancer incidence and mortality. In addition, there have been secular changes in cancer screening strategies (including colorectal and prostate cancer) and treatment since the study's inception. Moreover, we studied only cancer mortality and not non-fatal cancer rates. Taken altogether, these issues should be carefully considered in the interpretation of our results.

Our population consisted of asymptomatic healthy males employed by the Paris Civil Service. Socioeconomic status, prevalence of smoking, extent of alcohol use, and other factors might differ from the general population. Therefore, the extent to which the present findings can be generalized to a more unselected or recent population is unclear. The present results were observed after statistical adjustment for level of physical activity at inclusion. We have no data related to physical activity prior to enrollment or as a child or youth.

In summary, we have found a significant, strong and graded relationship between increased resting heart-rate, heart rate changes during exercise and over time and cancer mortality in a large population-based sample of middle-aged men. Although confirmatory studies are required and the mechanisms for such an association are unknown, heart-rate parameters may prove to be a simple and non-invasive stratification tool for the subsequent risk of malignant disease.
